# Higher incidence of premenopausal breast cancer in less developed countries; myth or truth?

**DOI:** 10.1186/1471-2407-14-343

**Published:** 2014-05-19

**Authors:** Reza Ghiasvand, Hans-Olov Adami, Iraj Harirchi, Rahim Akrami, Kazem Zendehdel

**Affiliations:** 1 Cancer Research Center, Cancer Institute of Iran, Tehran University of Medical Sciences, Tehran, Iran; 2 Department of Biostatistics, Institute of Basic Medical Sciences, University of Oslo, Oslo, Norway; 3 Department of Epidemiology, Harvard School of Public Health, Boston, MA, USA; 4 Department of Medical Epidemiology and Biostatistics, Karolinska Institutet, Stockholm, Sweden; 5 Sabzevar University of Medical Sciences, Sabzevar, Iran

**Keywords:** Breast cancer, Age-standardized rate, Risk factor, Annual percent change, Less developed countries, Premenopausal

## Abstract

**Background:**

Fundamental etiologic differences have been suggested to cause earlier onset of breast cancer in less developed countries (LDCs) than in more developed countries (MDCs). We explored this hypothesis using world-wide breast cancer incidence data.

**Methods:**

We compared international age-standardized incidence rates (ASR) of pre- (<50 years) and postmenopausal (≥50 years) breast cancers as well as temporal trends in ASRs of pre-and postmenopausal breast cancer among selected countries during 1975–2008. We used joinpoint log-linear regression analysis to estimate annual percent changes (APC) for premenopausal and postmenopausal breast cancer in the northern Europe and in Black and White women population in the US.

**Results:**

Premenopausal breast cancers comprised a substantially higher proportion of all incident breast cancers in LDCs (average 47.3%) compared to MDCs (average 18.5%). However, the ASR of premenopausal breast cancer was consistently higher in MDCs (29.4/100,000) than LDCs (12.8/100,000). The ASR of postmenopausal cancer was about five-fold higher in the MDCs (307.6/100,000) than the LDCs (65.4/100,000). The APC of breast cancer in Denmark was substantially higher in postmenopausal (1.33%) than premenopausal cancer (0.98%). Higher incidence of breast cancer among the white than black women in the US was pertained only to the postmenopausal cancer.

**Conclusion:**

The substantial and consistent lower age-specific incidence of breast cancer in LDCs than in MDCs contradicts the theory of earlier onset. Demographic differences with fewer old women in LDCs and lower prevalence of risk factors of postmenopausal cancer are the most likely explanation to the lower mean age at diagnosis in these countries.

## Background

Breast cancer is the most common malignancy among women and the fifth cause of death due to cancer both in the less developed (LDCs) and more developed (MDCs) countries worldwide [1]. However, among women living in MDCs (except Japan), world-age-standardized incidence rates (ASR) are still more than 4-fold higher than in LDCs. In 2008, ASR was estimated to be 89.9 per 100,000 women-years in Western Europe, but only 19.3 in Eastern Africa [1,2]. Although the incidence rate of breast cancer has decreased in the US and many other developed countries since early 2000s [3], it has increased 50-100% in some Asian countries including India and China during the last two decades [4,5].

Epidemiologic studies often distinguish premenopausal from postmenopausal breast cancer [6,7]. Some reproductive risk factors have stronger associations with risk of postmenopausal breast cancers [8-10] and obesity increases the risk of postmenopausal but decreases the risk of premenopausal breast cancer [11,12]. Therefore, it is conceivable that a higher prevalence of known and unknown causal factors in LCDs could increase chiefly the incidence of early onset disease.

In LDCs, the average age of women diagnosed with breast cancer is about 10 years lower than in MDCs [13-15]. Although the younger age structure of the population in the LDCs may explain this finding [5,16], it could also reflect a birth cohort phenomenon arising due to a higher prevalence of risk factors for breast cancer – such as low parity and late age at first birth in the younger compared to the older generation [17-19]. In addition, however, researchers and clinicians have proposed that earlier onset of breast cancer reflects more fundamental etiologic differences between women in LDCs and MDCs that remain to be discovered [14,20-23]. We aimed to investigate these rival interpretations of the difference in mean age at diagnosis of breast cancer in LDCs compared to MDCs.

## Methods

We used data published in the GLOBOCAN 2008 by the International Agency for Research on Cancer (IARC) to compare the proportion and truncated world age-standardized rate (ASR) of premenopausal (i.e. <50 years) and postmenopausal (i.e. ≥50 years) breast cancer in different countries in 2008 [24]. Based on GLOBOCAN, we classified the countries into less developed and more developed. In each group, we estimated weighted truncated ASR (world population) for the five countries with the highest coverage of cancer registration in the latest version of the “Cancer in Five Continents CI5 Volume IX” [25]. Data from the United Nations Population Division [26] were used to demonstrate the age structure and difference in the proportion of women aged 20–49 and ≥50 years from total women population (0 to ≥100 years) in the selected countries.

In order to illustrate changes in the incidence rate of breast cancer over time, we graphed the annual proportions and ASRs of pre- and postmenopausal breast cancer for Denmark, Finland, Norway and Sweden because these countries have had high quality nationwide cancer registration for more than 50 years. Because the Danish cancer registry provided the oldest data, we used it to demonstrate temporal trends in the pre and post-menopausal breast cancer ratio and ASR from 1943 to 2008 in Denmark. Data for Denmark, Finland, Norway and Sweden was obtained from the NORDCAN project (version 5.2) [27].

We also compared temporal trends of pre- and postmenopausal breast cancer incidences among Black and White population in the US. Data for the US Black and White populations was obtained from the Surveillance, Epidemiology and End Results (SEER) program of the National Cancer Institute [28].

We hypothesized that the incidence of premenopausal breast cancer would be stable over time and that changes in lifestyle factor would mainly affect the postmenopausal cancers. We used joinpoint log-linear regression analysis [29] to estimate annual percent changes (APC) for premenopausal and postmenopausal breast cancer. For trend analysis, the joinpoint regression allows to more accurately interpret changes over time, and also to determine if those changes are statistically significant. We used Monte Carlo permutation test to select the best fitting model. Joinpoint program (version 4.0) was used for statistical analyses [30]. All ASRs were standardized using the world standard population [31], and the weights were taken from the population distribution of the world standard population. The age group < 50 years and the age group ≥ 50 years defined as premenopausal and postmenopausal respectively [32].

## Results

The proportion of all breast cancer cases diagnosed before age 50 was substantially higher (47.3% in average) among the LDCs compared to the MDCs (18.5% in average). Specifically, 45.7%, 48.4% and 56.9% of breast cancer patients were diagnosed before age 50 in China, India and Algeria, whilst corresponding proportions were 21.5%, 19.1% and 15.9% in Australia, UK and Denmark. However, the average ASR for premenopausal breast cancer was lower in the LDCs (12.8 per 100,000) compared to the MDCs (29.4 per 100,000). Particularly, ASRs of premenopausal breast cancer were 12.1, 12.2 and 17 per 100,000 in China, India and Algeria, while corresponding ASRs in Australia, UK and Denmark were 30, 31.7 and 31.5 per 100,000 respectively (Table 1).

**Table 1:** **Estimated proportion and age standardized incidence rate (ASR) of breast cancer per 100,000 women-year in LDCs and MDCs in 2008 (GLOBOCAN 2008)**[24]

**Country**	**Overall**		**< 50 years old (premenopausal)**			≥ **50 years old (postmenopausal)**		
	**Number**	**ASR (W)**	**Number**	**Percent**	**ASR (W)**	**Number**	**Percent**	**ASR (W)**
**Less developed countries**								
China	169452	21.6	77449	45.7	12.1	92003	54.3	59.8
India	115251	22.9	55770	48.4	12.2	59481	51.6	65.3
Algeria	4271	28.6	2432	56.9	17	1839	43.1	75.2
Pakistan	19271	31.5	9878	51.3	16.6	9393	48.7	90.7
Egypt	12621	37.3	6152	48.7	20	6469	51.3	106.4
Weighted Average^*^		**23.3**		**47.3**	**12.8**		**52.7**	**65.4**
**More developed countries**								
Germany	64147	81.7	11289	17.6	28.8	52858	84.4	293.6
Canada	22986	83.2	4232	18.4	26	18754	81.6	311.9
Australia	13384	84.7	2877	21.5	30	10507	78.5	303.7
United Kingdom	46458	89.1	8866	19.1	31.7	37592	80.9	318.7
Denmark	4696	101.1	748	15.9	31.5	3948	84.1	379.4
Weighted Average		**85.1**		**18.5**	**29.4**		**81.5**	**307.6**

^*^The weights were taken from population distribution of the world standard population.

**Figure 1:**
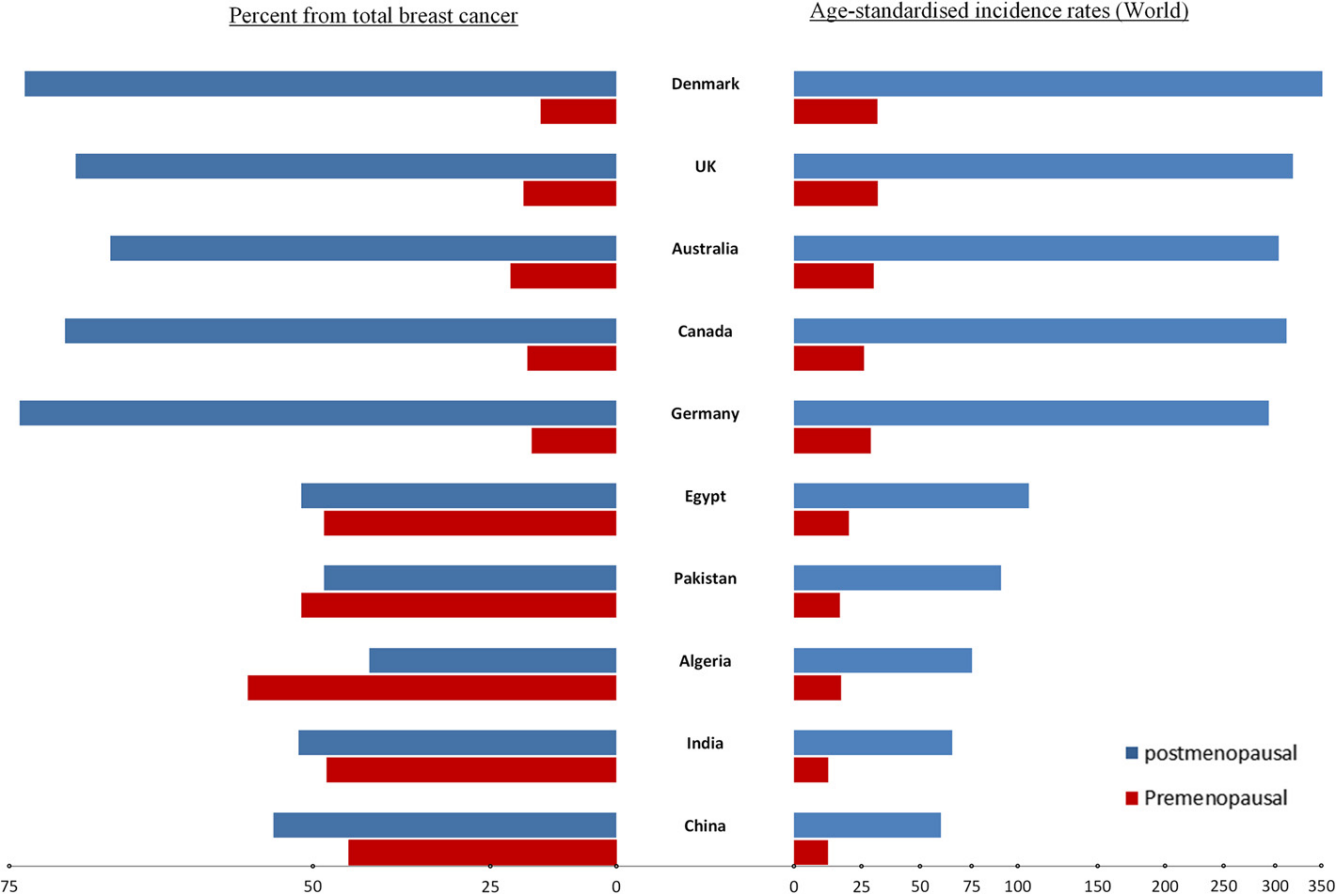
**Estimated proportions and age standardized incidence rate (ASR) of pre- and postmenopausal breast cancer on a log scale for selected countries in 2008 (GLOBOCAN 2008)**[16]**.**

**Figure 2:**
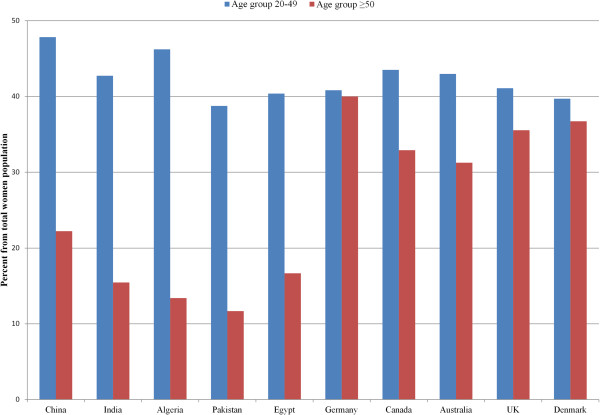
**Estimated proportion of Women population in age groups 20–49 and ≥50 from total women population in selected countries in 2005 (United Nations 2012) [**[26]**].**

The age-specific incidence rate of premenopausal breast cancer was almost similar in the LDCs and MDCs, being slightly higher in MDCs. However, incidence rate of postmenopausal breast cancer was substantially higher in the MDCs compared to the LDCs (Figure 1). The proportion of postmenopausal women was higher in MDCs compared to LDCs (Figure 2).

**Figure 3:**
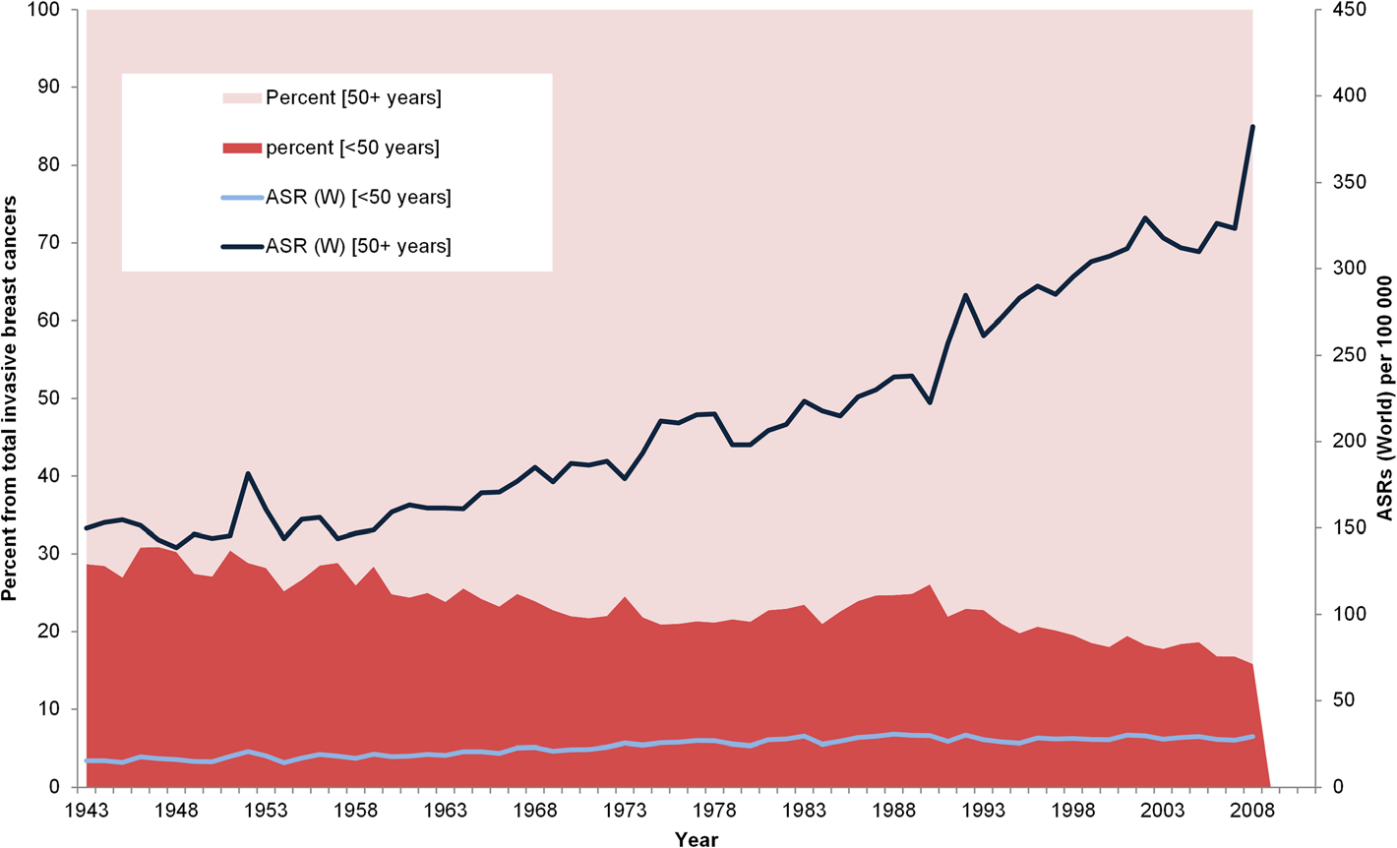
Premenopausal and postmenopausal breast cancer age standardized incidence rate (ASRs) and percent of premenopausal patients in Denmark from 1943 to 2008.

We found a dramatic increase in the ASR of postmenopausal compared to premenopausal breast cancer in Denmark from 1943 to 2008 (Figure 3). The annual percentage change for postmenopausal cancer was 1.33% (Table 2), and ASR increased 155% from 149.8 in 1943 to 382.4 per 100,000 in 2008. During the same period premenopausal breast cancer increased 91% from 15.3 per 100,000 in 1943 to 29.2 per 100,000 in 2008 (APC 0.98). Meanwhile, the proportion of premenopausal to all breast cancers decreased from 29% to 16% (APC -0.74).

**Table 2:** Annual percent change (APC) with 95% confidence intervals (CIs) in ASRs and proportion of premenopausal and ASR of postmenopausal breast cancer in Denmark from 1943 to 2008

**Premenopausal (< 50 years)**			**Postmenopausal (≥ 50 years)**	
	**Period**	**APC (95% CI)**	**Period**	**APC (95% CI)**
ASR*				
	1943 – 1986	1.54 (1.36 to 1.72)	1943 – 1958	0.18 (-0.42 to 0.78)
	1986 – 2008	-0.10 (-0.47 to 0.26)	1958 – 1985	1.45 (1.18 to 1.71)
	AAPC**	0.98 (0.81 to 1.15)	1985 – 2008	1.97 (1.64 to 2.29)
			AAPC	1.33 (1.13 to 1.54)
Proportion***				
	1943 – 1978	-1.04 (-1.21 to -0.88)	1943 – 1978	0.35 (0.30 to 0.41)
	1978 – 1988	1.48 (0.19 to 2.79)	1978 – 1988	-0.44 (-0.87 to -0.01)
	1988 – 2008	-2.04 (-2.42 to -0.64)	1988 – 2008	0.54 (0.41 to 0.67)
	AAPC	-0.74 (-0.84 to -0.64)	AAPC	0.29 (0.21 to 0.37)

^*^Age standardized incidence rate adjusted to the world standard population.

^**^Average annual percent change from 1943 to 2008.

^***^Proportion from total number of invasive breast cancers.

**Figure 4:**
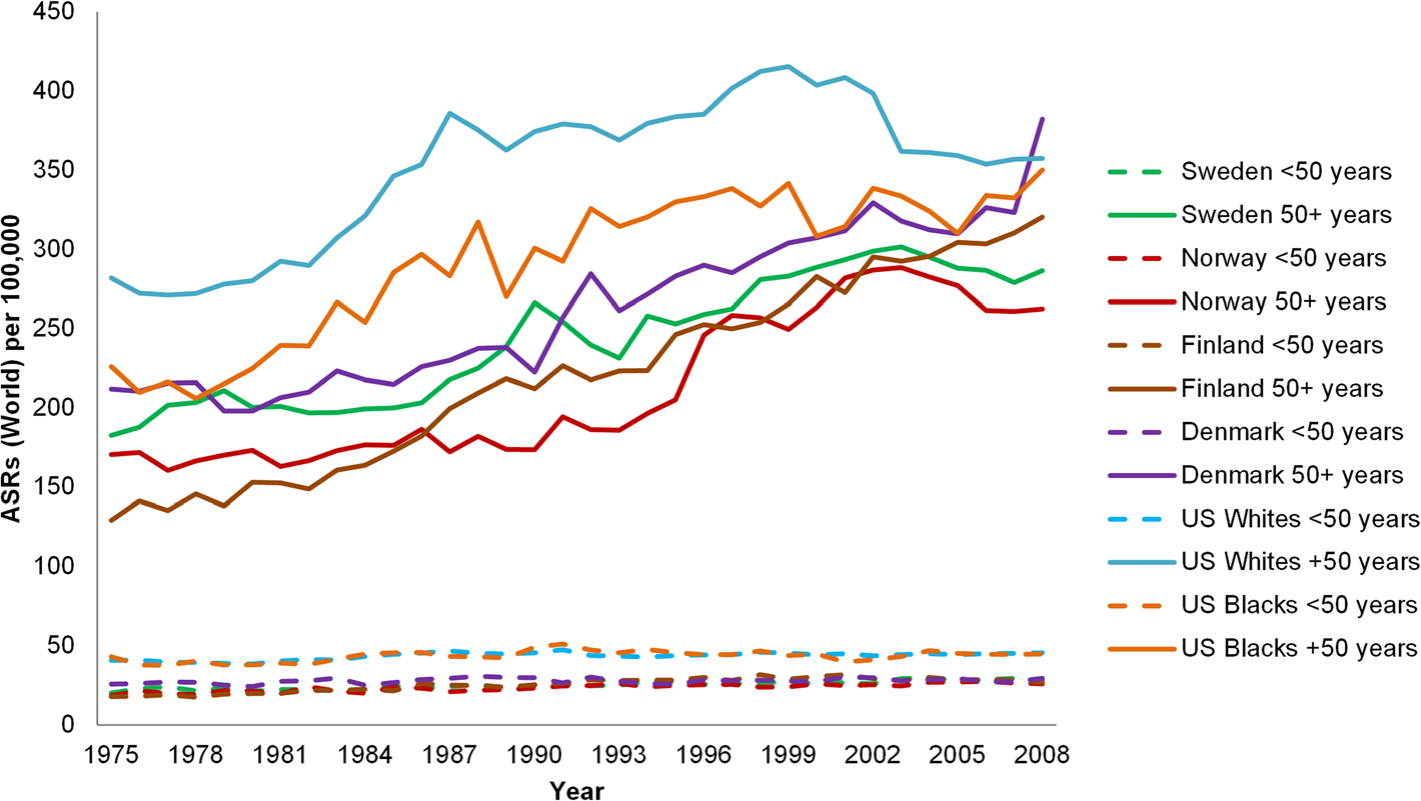
**Trend of pre- and postmenopausal incidence rates of breast cancer during 1975 to 2008.** Rates are age-adjusted to the world standard population. Sources: [27,28].

Although the incidence rates of premenopausal breast cancer increased slightly in all selected countries from 1975 through 2008, more significant changes happened in postmenopausal breast cancer incidence rate over time (Table 3). Furthermore, difference in the incidence rate of breast cancer between countries was considerable for postmenopausal breast cancer (Figure 4). Likewise, the difference in the risk of breast cancer among the black and white women pertained only to the postmenopausal women and the incidence of premenopausal women was equally low among them from 1975 to 2008.

**Table 3:** Annual percent change (APC) with 95% confidence intervals (CIs) in premenopausal (< 50) and postmenopausal (≥ 50) breast cancer age standardized incidence rate (ASRs), estimated with the best-fitting joinpoint model

**Country**	**Postmenopausal (≥ 50)**		**Premenopausal (< 50)**	
	**Period**	APC^* ^**(95% CI)**	**Period**	APC^* ^**(95% CI)**
Denmark				
	1975 – 1980	-1.23 (-4.00 to 1.63)	1975 – 2008	0.25 (0.05 to 0.46)^**^
	1980 – 2008	2.01 (1.80 to 2.23)^**^		
Sweden				
	1975 – 1979	3.00 (1.44 to 4.59)^**^	1975 – 2008	0.94 (0.79 to 1.10)^**^
	1979 – 1985	-1.10 (-2.17 to -0.03^**^		
	1985 – 1990	5.82 (4.22 to 7.45)^**^		
	1990 – 1993	-2.36 (-6.96 to 2.47)		
	1993 – 2002	2.63 (2.09 to 3.17)^**^		
	2002 – 2008	-1.30 (-2.10 to -0.49)^**^		
Finland				
	1975 – 1984	2.32 (1.51 to 3.13)^**^	1975 – 1998	2.48 (2.18 to 2.77)^**^
	1984 – 1988	6.26 (1.75 to 10.96)^**^	1998 – 2008	-1.20 (-2.19 to -0.19)^**^
	1988 – 2008	2.24 (2.00 to 2.84)^**^		
Norway				
	1975 – 2003	0.62 (0.30 to 0.94)^**^	1975 – 2008	0.96 (0.77 to 1.16)^**^
	1993 – 1997	8.08 (2.83 to 13.60)^**^		
	1997 – 2003	2.24 (-0.01 to 4.55)		
	2003 – 2008	-2.21 (-4.37 to -0.01)^**^		
US (whites)				
	1975 – 1982	0.89 (0.15 to 1.64)^**^	1975 – 1980	-1.09 (-2.48 to 0.32)
	1982 – 1987	5.54 (3.70 to 7.41)^**^	1980 – 1986	2.63 (1.18 to 4.09)^**^
	1987 – 1993	-0.27 (-1.50 to 0.98)	1986 – 2008	-0.07 (-0.22 to 0.08)
	1993 – 2000	1.69 (0.74 to 2.65)^**^		
	2000 – 2004	-3.79 (-6.42 to -1.08)^**^		
	2004 – 2008	-0.17 (-1.91 to 1.60)		
US (blacks)				
	1975 – 1978	-2.65 (-9.10 to 4.26)	1975 – 1991	1.32 (0.72 to 1.92)^**^
	1978 – 1986	4.64 (2.74 to 6.58)^**^	1991 – 2008	-0.43 (-0.97 to 0.12)
	1986 – 2008	0.67 (0.34 to 1.00)^**^		

^*^The model was specified to include a maximum of six joinpoits. The model constrained the joinpoints to be at least five years from each other (to be more conservative) and from the boundaries of the total study period.

^**^Statistically significant annual percent changes are printed in bold.

## Discussion

We showed that the incidence rate of premenopausal breast cancer in the LDCs is indeed invariably lower than that in the MDCs. However, the proportion of premenopausal breast cancer from total breast cancers is substantially higher in these countries compared to MDCs. In addition, our findings reveal that the global variation and increase in the incidence rate pertains mostly to postmenopausal breast cancers.

In LDCs, the majority of breast cancers occur among premenopausal women and the mean age of patients is around 50 years. Some investigators have concluded that breast cancer occurs about one decade earlier in several populations including Iran [23], Mexico [14], Africa [20] and blacks in the UK [21]. Some have also suggested the existence of specific genetic and environmental risk factors in LDCs [4,14] which would call for specific prevention and early detection strategies in these countries such as starting mammography screening already at age 40 [13]. However, these conclusions were based on comparison of median age and proportion of breast cancer in different age groups rather than a comparison of age-specific incidence rates in LDCs and MDCs.

International variations in coverage and accuracy of cancer registries and diagnostic/screening strategies might explain part of the dramatic difference in the incidence rate of postmenopausal breast cancer worldwide. About one fourth of postmenopausal breast cancer in MDCs with national mammography screening is attributed to overdiagnosis [33,34]. In a comparative study between Sweden and Singapore, both with nationwide cancer registries, difference in incidence of breast cancer was shown to be unrelated to differences in registration systems. Hence, cohort effects due to changes in lifestyle and reproductive factors in these countries as well as the screening impact need to be taken into account [18,35]. Variations in lifestyle and reproductive risk factors seem to play a more important role in the global differences in the incidence of postmenopausal breast cancer. In several studies [19,35-37], the increasing incidence of breast cancer follows changes in the reproductive and lifestyle factors.

Some established risk factors including age at menarche and age at menopause, include hormonal mechanisms, which are involved in the development of breast cancer [38]. Estrogen receptor (ER), progesterone receptor (PR), and human epidermal growth factor receptor 2 (HER2), are the tumor markers that have been widely studied in relation to the etiology, prognosis and treatment of breast cancer subtypes [8,38,39]. Some epidemiological studies have reported heterogeneity of breast cancer risk factors with hormone receptors and the expression of HER2 [40]. Reproductive factors and BMI were shown to be associated with ER + and PR + breast cancer, compared with ER- and PR- tumors [8,41]. The prevalence of hormone receptor negative cancer is higher among premenopausal women than among postmenopausal women, while ER + and PR + breast cancer in more prevalent after menopause and its incidence increases with age [42,43].

The relatively stable incidence rate of premenopausal breast cancer during several decades [44,45] suggest a predominant role of genetic and constant environmental risk factors. Moreover, several studies showed that only postmenopausal breast cancer risk increased among women who moved from low risk to high-risk countries [10,46-48]. In a recent study among migrant women in Sweden, risk of breast cancer increased only at postmenopausal ages [10]. A study of migration history and risk of breast cancer showed a significant increase in the trend of postmenopausal breast cancer risk in the second and third generation of Hispanic immigrants, but not in premenopausal breast cancer, after adjusting for lifestyle and reproductive factors [49]. In addition, a study of racial/ethnic differences in the incidence of postmenopausal breast cancer in the Women’s Health Initiative revealed significant ethnic differences, with lower incidence among Asian/Pacific Islanders, African American and Hispanics compared with Whites. However, after adjustment for established risk factors, the observed differences were attenuated and statistically not significant [45].

## Conclusions

The global variation in the incidence rate of breast cancer is due chiefly to the differences in the risk of postmenopausal breast cancer and variation in exposure to the reproductive and lifestyle risk factors. Aging of the population and adaptation of western lifestyle in the LDCs would soon lead to a higher incidence of breast cancer in these countries. While the prevention and policy making at the time being should be focused on the bulk of young breast cancer patients in LDCs, it is essential to take a closer look and monitor the transition to higher incidence of postmenopausal breast cancer in the future and adapt the preventive and cancer control policies accordingly.

## Abbreviations

ASR: Age-standardized rate; LDCs: Less developed countries; MDCs: More developed countries; APC: Annual percent change; ER: Estrogen receptor; PR: Progesterone receptor.

## Competing interest

The authors declare that they have no competing interests.

## Authors’ contributions

RG participated in the study design, performed statistical analysis and drafted the manuscript. HOA contributed in the interpretation of data and critically revised the manuscript for important intellectual concept. IH participated in the study design and interpretation of data. RA participated in the study design and interpretation of data. KZ conceived the study and contributed in the interpretation of data and helped draft the manuscript. All authors read and approved the final manuscript.

## Pre-publication history

The pre-publication history for this paper can be accessed here:

http://www.biomedcentral.com/1471-2407/14/343/prepub
